# Gut Microbiome Regulation of Gut Hormone Secretion

**DOI:** 10.1210/endocr/bqaf004

**Published:** 2025-03-03

**Authors:** Jessica Chao, Rosemary A Coleman, Damien J Keating, Alyce M Martin

**Affiliations:** Gut Hormones in Health and Disease Lab, Flinders Health and Medical Research Institute, College of Medicine and Public Health, Flinders University, Adelaide 5042, Australia; Gut Hormones in Health and Disease Lab, Flinders Health and Medical Research Institute, College of Medicine and Public Health, Flinders University, Adelaide 5042, Australia; Gut Sensory Systems Group, Flinders Health and Medical Research Institute, College of Medicine and Public Health, Flinders University, Adelaide 5042, Australia; Gut Hormones in Health and Disease Lab, Flinders Health and Medical Research Institute, College of Medicine and Public Health, Flinders University, Adelaide 5042, Australia

**Keywords:** enteroendocrine cells, gut microbiome, gut hormone, host physiology

## Abstract

The gut microbiome, comprising bacteria, viruses, fungi, and bacteriophages, is one of the largest microbial ecosystems in the human body and plays a crucial role in various physiological processes. This review explores the interaction between the gut microbiome and enteroendocrine cells (EECs), specialized hormone-secreting cells within the intestinal epithelium. EECs, which constitute less than 1% of intestinal epithelial cells, are key regulators of gut–brain communication, energy metabolism, gut motility, and satiety. Recent evidence shows that gut microbiota directly influence EEC function, maturation, and hormone secretion. For instance, commensal bacteria regulate the production of hormones like glucagon-like peptide 1 and peptide YY by modulating gene expression and vesicle cycling in EE cells. Additionally, metabolites such as short-chain fatty acids, derived from microbial fermentation, play a central role in regulating EEC signaling pathways that affect metabolism, gut motility, and immune responses. Furthermore, the interplay between gut microbiota, EECs, and metabolic diseases, such as obesity and diabetes, is examined, emphasizing the microbiome's dual role in promoting health and contributing to disease states. This intricate relationship between the gut microbiome and EECs offers new insights into potential therapeutic strategies for metabolic and gut disorders.

Over 10^4^ commensal gut microbiota, viruses, fungi, and bacteriophages form one of the largest microbial ecosystems in the human body—the gut microbiome (1). The bacterial abundance, diversity and overall composition of the microbiome varies along the length of the digestive tract, with the largest populations found in the large intestine (2). A major function of gut bacteria is to aid in the digestion of nutrients along the gut. However, there is clear evidence that the gut microbiome regulates and may play a casual role in various host physiological processes. These include gut–brain axis communication (satiation and satiety) (3), gut motility (4), energy metabolism (5), and mental health (6).

The microbiome interacts closely with hormone-secreting enteroendocrine cells (EECs) embedded along the intestinal epithelium (7). The gut epithelium is a dynamic barrier consisting of multiple cell types that are essential in facilitating the uptake of nutrients while maintaining overall gut function (8). EECs and the hormones they produce are on the front-line of interactions between the gut and the internal environment. EECs are a heterogeneous cell population that produce and secrete up to 20 different hormones, which act locally on surrounding epithelial cells in a paracrine manner, or directly simulate vagus nerve endings at the site of hormone release [8]. Hormone release from EECs stimulate different neural pathways by acting on nerve endings of intrinsic and extrinsic neurons. These include extrinsic pathways along the gut–brain axis which control appetite and satiation, as well as intrinsic pathways within the gut's enteric nervous system that control gut function. Alterations in physiological gut functions may also play a role in the development of enteric gut disorders like hyperphagia, function dyspepsia, gastroparesis, and irritable bowel syndrome. Once secreted into circulation, enteroendocrine (EE) hormones can also stimulate various target tissues and organs to regulate host metabolism, digestion, gut motility, gastric emptying, and overall gut health (7).

EECs can sense gut bacteria and their metabolites via receptors expressed on the apical membrane (Fig. 1). Interactions between the microbiome and EECs are complex and are mediated by toll-like receptors (TLRs) (9), olfactory receptor 558 (Olfr588), (10) irritant receptor (TRPA1) (11), taste receptors (12), free fatty acid receptors (FFARs) (FFAR1, FFAR3, FFAR2, and FFAR4), and G protein–coupled receptors (GPR119) (13) (Fig. 2). The everchanging nature of the gut microbiota due to diet and lifestyle factors can also alter the composition and abundance of their metabolites, which affects the diversity of hormones secreted by the heterogeneous EEC population.

**Figure 1.**
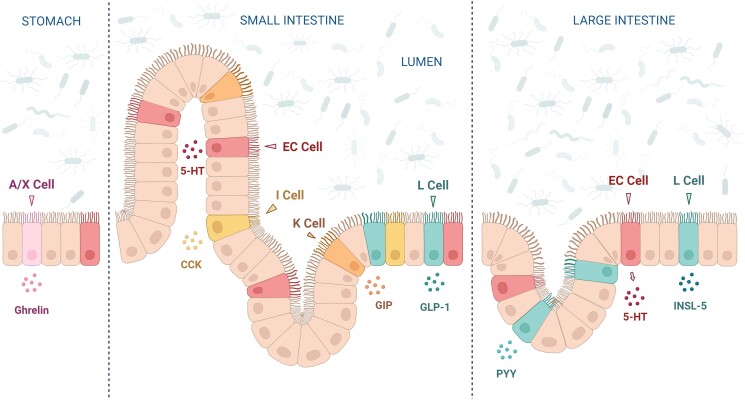
Enteroendocrine (EE) hormone secretion along the gastrointestinal tract. Enteroendocrine cells (EECs) reside in the epithelium, making direct contact with dietary components and microbial products within the gut lumen. The luminal contents trigger the expression and secretion of different gut peptides, such as serotonin (5-HT); cholecystokinin (CCK); glucose-independent insulinotropic peptide (GIP); insulin-like peptide 5 (INSL-5); peptide YY (PYY); glucagon-like peptide 1 (GLP-1).

**Figure 2.**
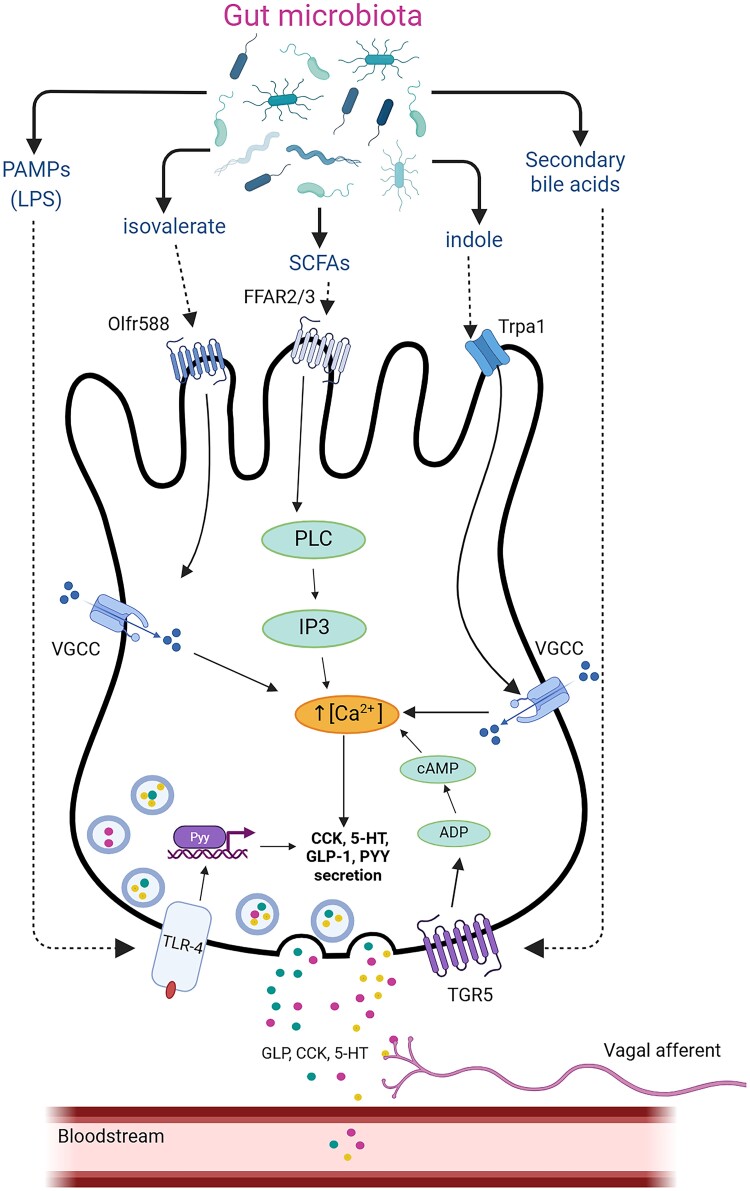
Gut microbiota interactions with EECs. The gut microbiota generates a range of metabolites through the fermentation or enzymatic breakdown of proteins, carbohydrates, and primary bile acids. EECs sense microbial cues from the gut lumen through receptors, such as FFARs and Olfr588, which are mostly activated by fatty acids, including short-chain fatty acids (SCFAs) and branched-chain fatty acids. Secondary bile acids can signal through the Takeda G-protein receptor 5 (TGR5) on the basolateral membrane and through farnesoid X receptor (FXR), both of which are abundantly expressed in EECs in the distal gut. Indole, a catabolite derived from tryptophan, influences intestinal motility by activating transient receptor potential ankyrin A1 (Trpa1) on 5-HT expressing EC cells which can trigger calcium entry via voltage-gated calcium channels (VGCC). EECs directly sense bacteria via toll-like receptors (TLRs), which recognize pathogen-associated molecular patterns (PAMPs) that are unique to each microorganism.

In this review, we will examine the mechanisms that underpin the critical interactions between the gut microbiome and EECs. We will explore the current literature on how the gut microbiome regulates the functional biology of EECs in the context of bacterial metabolites, direct bacterial signaling, and viral contribution. Furthermore, we will discuss the wide-reaching implications that these microbiome–EEC interactions have for human health and disease.

## Enteroendocrine Cells

EECs exists as a diverse heterogeneous population of epithelial cells that are commonly characterized by their hormone profile and morphology (14, 15). Although EECs make up less than 1% of epithelial cells in the intestine, collectively they form the largest endocrine organ in the human body (16). We have focused our review on some of the most well characterized EECs which include, but are not limited to, ghrelin-secreting A (X-like) cells, cholecystokinin (CCK)-secreting I cells, glucose-dependent insulinotropic peptide (GIP)–secreting K cells, serotonin (5-HT)-secreting enterochromaffin (EC) cells, and glucagon-like peptide 1 (GLP-1), glucagon-like peptide 2 (GLP-2), and peptide YY (PYY)–secreting L cells (17). In previous dogma, the nomenclature of each EEC subtype has been inferred by the principal hormone they produce (17). However, mounting evidence suggests that each EEC subtype produces a combination of different hormones simultaneously throughout their lifespan (18, 19). As frontline sensory cells within the gastrointestinal (GI) tract, EECs are polarized cells that can detect and respond to changes in the intestinal environment via receptors located on apical and basolateral membrane (7). Single cell gene expression analysis reveal an enrichment of bacterial metabolite receptors in EC cells (10, 20), as well as nutrient receptors that are expressed differentially between the small intestine and colon (21). The mechanisms by which the gut microbiota regulates EEC sensing and its secretome will be explored in greater depth below.

## Microbial Regulation of Enteroendocrine Cells

Other than aiding the digestion of food, the microbial system also influences the activity of EECs. Recent evidence from transcriptomics analysis revealed that commensal microbiota colonization significantly increased the expression of genes associated with mitochondrial function (22). They found that germ-free zebrafish lacked the necessary mitochondrial function required for EECs to respond to nutrient stimulation, a key characteristic in EEC maturation (22). This indicates that the presence of bacteria is essential for EEC maturation, although the mechanism by which bacteria regulates mitochondrial function is yet to be defined. Dietary changes can also act directly via the gut microbiome to alter EEC sensing. In zebrafish, high-fat feeding altered the morphology of EECs and reduced its capacity to respond to glucose and palmitate stimulation, a phenomenon known as EEC silencing (23). However, in germ-free zebrafish, EEC silencing is abolished after high-fat feeding, suggesting that the microbiota is necessary to convey the effects of diet on EEC morphology and function (23). Germ-free murine studies indicate that gut bacteria modulate L cell density and peptide secretion, where gut bacteria are most abundant and GLP-1 and PYY expression is the highest (24-26). It has been well documented in germ-free mice that plasma levels of GLP-1 and PYY is higher than conventionally raised mice (25). Transcriptomic profiling of L cells derived from a Gcg reporter mouse showed that microbiota significantly altered L cell gene expression in the ileum compared to germ-free counterparts (24). Gene ontology enrichment analysis revealed that gut microbiota suppressed genes related to vesicle localization and cycling in L cells, which was further supported by a reduction in vesicle numbers and intracellular GLP-1 in conventionally raised mice (24). Aside from GLP-1, other EE hormones, such as 5-HT, is also regulated by microbiota. Germ-free animals have significantly lower levels of plasma 5-HT and reduced tryptophan hydroxylase 1 (Tph1) expression, a rate-limiting enzyme involved in the biosynthesis of 5-HT from tryptophan (27). They demonstrated that the presence of indigenous spore-forming bacteria, mainly from the *Clostridial* species, increases 5-HT biosynthesis through increasing Tph1 expression (28). Collectively, these studies indicate that the microbiota is necessary for proper EE function and maturation, as well as the production and secretion of EE peptide hormones in response to nutritional cues from the gut environment.

EECs possess a unique microbial sensing repertoire that is distinct from other gut epithelial cell types (10). There is a plethora of evidence demonstrating bacterial sensing in EECs, including the detection of microbial metabolites and pathogen-associated molecular patterns from bacteria (Fig. 2). The metabolites with demonstrated bioactivity on EECs include short-chain fatty acids (SCFAs), lipopolysaccharides, indole, and secondary bile acids. In addition to these metabolites, EECs also express olfactory and irritant receptors that convey messages from the gut epithelium to afferent nerve endings, thus allowing the host to respond to luminal changes in the gut by stimulating physiologic responses such gut motility and visceral pain (10). The role of these receptors and their corresponding metabolite ligands will be examined in greater detail to determine their potential impact on host physiology.

## Short Chain Fatty Acids

SCFAs are end-products of carbohydrate and dietary fiber fermentation by anaerobic bacteria in the large intestine (29). Firmicutes and Bacteroidetes produce most of the SCFAs (30), including acetate, butyrate and propionate that exist in at a ratio of 3:1:1 in the human intestinal lumen, respectively (31). These bacterial species express carbohydrate-active enzymes which humans lack, enabling them to break down dietary fibers, which would otherwise not be absorbed (29). SCFAs are also associated with regulatory roles in metabolism. Acetate, produced by firmicutes, is used readily in lipid metabolism (32), while propionate, derived from bacteroidetes, supports gluconeogenesis (33) and inhibits cholesterol synthesis (34). Butyrate, predominantly produced by firmicutes, is the primary energy source for colonocytes and regulates their proliferation and differentiation along the gut epithelium (35).

In addition to these roles, SCFAs also act as signaling molecules that bind to FFAR2 and FFAR3, which are cell surface G-protein–coupled receptors expressed in a number of tissues including the intestine (36). Within the gut, FFAR2 and FFAR3 are implicated in multiple processes such as inflammation (37, 38), feeding behavior (39), gut integrity (40), and hormone synthesis (41-48). Quantitative PCR analysis of the intestinal mucosa reveal that *Ffar2* and *Ffar3* gene expression are enriched in human (46, 49), mouse (37, 50), and rat (51) EECs. FFAR2 and FFAR3 expression were further confirmed at the protein level in reporter mice fluorescently tagged with monomeric red fluorescent protein (mRFP) (52). FFAR3-mRFP was strongly expressed in GLP-1 and PYY cells within the proximal colon, while FFAR2-mRFP reporter was expressed in leukocytes in the lamina propria but only weakly expressed in EECs in the small intestine (37). FFAR2 stimulation by SCFAs induces GLP-1 secretion in a calcium-dependent manner in primary murine colonic L cells (48). EC cells also express *Ffar2* and *FFar3*; however, acute stimulation with either acetate, butyrate, or propionate does not trigger hormone release (53). Rather, long-term exposure of SCFAs increases the production of 5-HT via increased expression of *Tph1*, as shown in the EC-like cell line, RIN14B (28). mRNA expression of chromogranin A (*Chga*), an EEC marker, was increased in human and mouse enteroids when exposed to butyrate, but not acetate or propionate (45). Exposure to butyrate also stimulated PPY and ghrelin secretion from 2D human enteroids (45). Mice lacking *Ffar2* exhibited reduced mRNA levels of glucagon (*Gcg*), a precursor to GLP-1, PYY, and active GLP-1 peptide (48). Additionally, both *Ffar2*^−/−^ and *Ffar3*^−/−^ mice have impaired glucose tolerance, and elevated fasting and postprandial blood glucose levels compared with wild-type mice, supposedly due to reduced GLP-1 and PYY production (48). Germ free *Ffar2*^−/−^ mice that were cocolonized with *Bacteroides thetaiotaomicron* and *Methanobrevibacter smithii* (known producers of SCFAs) had much lower levels of fasting serum PYY levels than gnotobiotic *Ffar2*^+/+^ mice (54). Since PYY inhibits gut motility in mice, gnotobiotic *Ffar2*^−/−^ mice exhibited increase rate of intestinal transit, which suggests that FFAR2 can regulate gut motility via direct activity on EECs (54). Oral administration of butyrate in lean mice increased plasma PYY, and total and active plasma GLP-1, while both butyrate and propionate triggered an increase in GIP levels (42). Intracolonic administration of propionate stimulates the release of both GLP-1 and PYY in rats; however, in *Ffar2*^−/−^mice, propionate-induced hormone release was attenuated (47). *Ffar3*^−/−^ mice have attenuated levels of GLP-1 release after butyrate administration, suggesting that FFAR3 is required for maximal GLP-1 induction by butyrate (42). FFAR3 does not appear essential for GIP release, as FFAR^−/−^ mice show normal GIP levels compared to wild-type mice (42).

Aside from SCFAs, branched-chain fatty acids, such as isovalerate, are also implicated in EEC signaling and hormone release. Unlike butyrate, acetate and propionate, branched-chain fatty acids are produced from microbial breakdown of protein as opposed to insoluble starches and carbohydrates (55). They also exist in smaller proportions in the distal gut, comprising 5% to 10% of total SCFAs (55). Isovalerate is an agonist for Olfr588, which is expressed in colonic EC cells according to qPCR analysis (56) and single cell RNA sequencing analysis (57) on mouse intestine. Interestingly, Olfr588 expression is only limited to EC cells within the colon and are not expressed by other epithelial and EEC types (56, 57), suggesting that ECs have a unique microbial sensing ability. They also found that a catalogue of olfactory receptors, namely OR73, hOR17-7/11, OR1G1, and hOR17-210, that are expressed in a human EC cell line (BON) (58). While informative, these studies do not indicate how isovalerate or other Olfr588 agonists trigger downstream signaling pathways that lead to consequent 5-HT release from ECs. A major study using ChgA-GFP tagged intestinal organoids showed that isovalerate activates Gα_olf/s_-adenylyl cyclase signaling cascade in EC cells, thus promoting intracellular Ca^2+^ influx via downstream voltage gated calcium channels (10). Consistent with this study, 1 group used Ca^2+^ imaging to show that human olfactory receptors stimulation with odorant ligands also triggered a Ca^2+^ influx, which led to 5-HT secretion (58). Intestinal organoids lacking Olfr688 gene were unable to evoke large Ca^2+^ responses from isovalerate, which demonstrates that Olfr588 is required for isovalerate signaling in EC cells (10). Isovalerate signaling through Olfr588 triggered the release of 5-HT, which further activated 5-HT_3_ receptor expressing primary afferent nerve fibers that interact in a synaptic-like manner with EC cells (10). This study clearly demonstrates a pathway by which gut microbes can indirectly communicate with the nervous system and provoke physiological responses, such as gut motility, and visceral pain.

## Secondary Bile Acids

Our understanding of bile acid function has evolved over the last decade from that of simple lipid emulsifiers to complex signaling molecules (59). Bile acids are synthesized from cholesterol in the liver and are released into intestine to aid the emulsification and absorption of lipids across the intestinal epithelium (60). In the liver, primary bile acids, cholic acid (CA) and chenodeoxycholic acid (CDCA), are chemically conjugated with either a glycine or taurine amide group, and stored in the gallbladder until it is released into the intestinal lumen (60). While most primary bile acids are absorbed by intestinal epithelial cells, a portion of conjugated bile acids become deconjugated by gut bacteria and thus form secondary bile acids, some of these include lithocholic acid (LCA) and deoxycholic acid (DCA) (60). Both primary bile acids and bacteria-derived secondary bile acids have demonstrated bioactivity on EECs.

Bile acids are potent stimulants of the farnesoid X receptor (FXR) (61) and Takeda G protein–coupled receptor 5 (TGR5) (62). Primary bile acids prefer to stimulate FXR (CDCA > CA > LCA > DCA), whereas secondary bile acids are more likely to be endogenous ligands for TGR5 activation (LCA > DCA > CDCA > CA) (63). TGR5 expression is not limited to the intestine, but is also expressed widely in the skeletal muscle, spleen, brown adipose, and the gallbladder (64). TGR5 expression is enriched within the human ileum and colon of EECs (65), which coincidently is where L cell density is the highest. Stimulation of TGR5 by bile acids increases GLP-1 and PYY secretion in GLUTag cells, a murine L cell line (66), while LCA individually stimulates L cell differentiation in mouse and human intestinal organoids (67). TGR5 is localized to the basolateral membrane of L cells, which suggests that bile acids must be absorbed, then released into the basolateral space, where they can bind to the receptor (68). In a perfused rat model, vascularly administered taurine conjugated deoxycholic acid (TDCA) was more effective in triggering GLP-1 secretion than TDCA that was administered via the lumen (68). The terminal ileum is the primary site of bile acid absorption and is facilitated by the apical sodium-dependent bile acid transporter (ASBT) (69, 70). One may suggest that bile acids in the ileum would have the maximum bioactivity on TGR5 compared with other regions of the gut, such as the colon, where ABST expression is significantly lower (70). When TDCA and TGR5 agonist were used to trigger GLP-1 release in rat intestinal cultures, it was found that GLP-1 secretion was significantly hindered in the presence of an ASBT inhibitor (68). Ileo-colonic delivery of conjugated bile acids in humans resulted in increased postprandial GLP-1 levels, as well as improved glucose tolerance (65).

Contrary to TGR5, binding to FXR inhibits GLP-1 secretion and production by downregulating *Gcg* expression (71). FXR activation interferes with the carbohydrate-responsive element binding protein, a transcription factor that is necessary for *Gcg* expression (71). FXR^−/−^ mice have upregulated proglucagon expression and increased GLP-1 secretion postprandially (71). Other studies have also shown that FXR^−/−^ mice are also protected against diet-induced weight gain, and elevated fasting glucose and insulin levels (72), perhaps due to an increase in the production and secretion of L cell hormones (73). In a diet-induced obesity mouse model, diverting bile flow from the gallbladder anastomosis to the ileum produces similar physiologic changes as Roux-en-y bariatric surgery, which includes sustained improvements in weight, glucose tolerance and hepatic steatosis (74). After Roux-en-y bariatric surgery, ingested food as well as bile bypasses a large portion of the small intestine, allowing increased delivery of dietary components and bile acids to the distal regions of the gut (75). This is also where the density of L cells is at the highest, and theoretically where more L cells can be stimulated, and more GLP-1 is released (76). In mice, bile acid diversion to the ileum increased luminal bile acid levels, particularly tauro-β-muricholic acid, a known FXR antagonist (74). It is thought that elevated levels of tauro-β-muricholic acid can reduce the activity of FXR signaling in the ileum, although FXR levels in treated mice are no different to that of the control (74). Most interestingly, levels of TGR5 are significantly reduced in the ileum after bile acid diversion (74), which suggests that mechanisms other than TGR5 and FXR may play role in the physiological outcomes of this procedure.

## Indole

Indole is the most abundant metabolite derived from the breakdown of tryptophan by gut bacteria (77). There are more than 85 gram-positive and gram-negative species known to produce indole, of which *Escherichia coli* is one of the most studied bacteria (77). Indole and other tryptophan derivates have well defined roles in intestinal barrier function and host immune homeostasis through ligand binding to the transcription factors, Ahr and Pxr, but they also serve as potent modulators of gut hormone release (78, 79). A study in mouse colonic L cell line (GLUTag) reported that indole triggered significant increase in GLP-1 secretion within 5 minutes of stimulation, while prolonged incubation with indole (240 minutes) greatly reduced its secretion (80). The stimulatory effect of indole is thought to be due to prolonged activation of voltage-gated Ca^2+^ channels, which is known to trigger the exocytosis of GLP-1 containing vesicles (80). They also found that indole acts as NADH inhibitor and prolonged exposure greatly reduced intracellular ATP concentration (80). ATP is required for opening ATP-sensitive K^+^ (K_ATP_) channels, therefore lower ATP concentration will limit membrane depolarization, and, consequently, reduce GLP-1 secretion (73). While this demonstrates that indole activates EECs and triggers hormone release, the receptor that mediates this effect is still unknown. Using real-time measurements of EEC and nerve activity in zebrafish, it was found that indole acts via the receptor transient receptor potential ankyrin A1 (Trpa1) to trigger the release of 5-HT (11). Previous studies have shown that Trpa1 is highly expressed in EC cells in humans, mice, and rats, and stimulation of EC cells with TRPA1 agonists, including allyl isothiocyanate and cinnamalde-hyde, increases intracellular Ca^2+^ levels and 5-HT release (81). They also showed that activating Trpa1 promotes the contraction of isolated guinea pig ileum via the 5-HT^3^ receptor, suggesting a functional role in regulating gut motility (81). Ye and colleagues were able to show that *Edwardsiella tarda,* a known producer of indole and other tryptophan derivates, was able to trigger 5-HT secretion from EECs through activation of Trpa1 (11). This resulted in increased intestinal motility due to the activation of stimulated vagal sensory ganglia and cholinergic enteric neurons by 5-HT (11). This demonstrates that the gut microbiota act via Trpa1^+^ EC cells to regulate GI function.

## Physiological Implications of the Gut Microbiome

**Figure 3.**
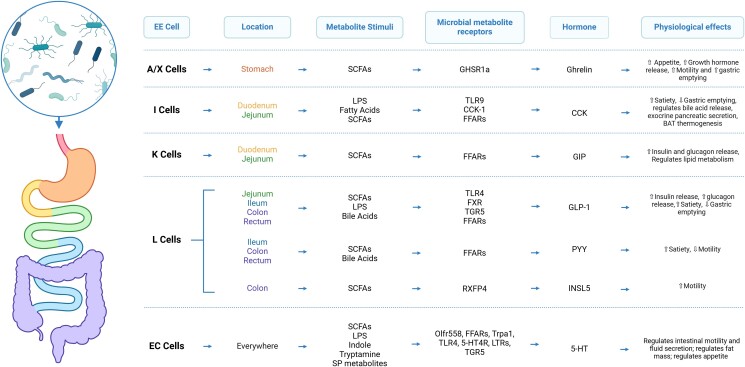
Microbial regulation of host physiology through EEC hormones. EECs have the capacity to interact with microbiota from multiple regions along the gastrointestinal tract, including the stomach, upper gut (duodenum, jejunum, ileum) and distal gut (colon, rectum). Microbial metabolites, such as SCFAs, bile acids and indole, are ligands that act on their corresponding receptor. Activating these receptors trigger the release of gut hormones, which directly regulate key physiological processes such as appetite control, gut motility, insulin release, brown adipose tissue (BAT) thermogenesis, and lipid metabolism.

Given the clear interactions between the microbiome and EECs, it is not surprising that these interactions profoundly influence many of the body's physiological processes (Fig. 3). Here we have focused broadly on just 3 of these processes: motility and gastric emptying, feeding behavior, and metabolism.

## Motility and Gastric Emptying

5-HT, ghrelin, and insulin-like peptide-5 stimulate and increase gut motility, whereas the anorexigenic hormones CCK, PYY, and GLP-1 inhibit it (82). It is clear that 5-HT effects on GI motility and gastric emptying (83, 84) are likely to be, in part, modulated by the microbiome. Metabolites derived from spore-forming bacteria raise 5-HT levels and increasing GI motility (28). EC cells express receptors such as Olfr558 and Trpa1 that respond to bacterial metabolites, triggering 5-HT release and an increase in GI motility (10, 11, 56). Luminal infusion of butyrate restores motility deficits in germ-free mice, an effect blunted in Tph1^−/−^ mice, suggesting ECs mediate these effects (85).

GLP-1 and PYY release in the distal intestine can influence gastric emptying, and pancreatic and biliary secretion in the proximal intestine. They do this by contributing to the “ileal brake”—a feedback mechanism that slows proximal GI motility, secretion, and increases feelings of satiety, reducing food intake (86). GLP-1 and PYY release is mediated by microbial metabolites including SCFAs, and primary and secondary bile acids. SCFAs upregulate L cell differentiation and density (43, 67, 87), which increases postprandial GLP-1 and PYY release and satiety. Conversely, some primary and secondary bile acids inhibit GLP-1 expression in L cells via the FXR (71, 88, 89). The conflicting roles that these receptors play on the same populations of EECs demonstrates the complex feedback loops that exist to regulate gut motility via gut hormone release.

## Feeding Behavior

Appetite control involves satiety cues derived from the gut which are sent to the brain, driving behaviors that affect eating (90). Bacteria and their metabolites activate EEC receptors, prompting hormone secretion either into systemic circulation or the lamina propria to stimulate local vagal afferent nerves (91). PYY, GLP-1, and CCK are anorexigenic hormones responsible for inducing satiety postprandially (92, 93). Ghrelin is an orexigenic hormone produced in abundance in the stomach and is released during fasting periods to increase appetite and food-seeking behaviors (94). The hypothalamic arcuate nucleus (ARC), and the solitary nucleus and area postrema in the brainstem form the primary centers for appetite regulation in the mammalian brain (95). Within the ARC, feeding behavior is regulated by 2 distinct neuronal populations: orexigenic agouti-related peptide (AGRP) neurons and anorexigenic proopiomelanocortin (POMC) neurons (95). Ghrelin activates AGRP neurons to drive food seeking behaviors (95). After food intake, GLP-1 and PYY enter systemic circulation directly stimulating receptors in the ARC or via the vagus nerve (96-98). PYY_3-36_ can cross the blood–brain barrier and inhibit AGRP neurons in the ARC by binding to Y2 receptors to induce satiety (99) through disinhibiting neighboring POMC neurons, thereby activating a satiety pathway to reduce food intake (100). GLP-1 elicits its satiety effects by binding on GLP-1R expressed on POMC neurons and indirectly via GABAergic neurons to inhibit AGRP neurons (101).

It is believed that increasing the production of SCFAs through bacterial fermentation of nondigestible carbohydrates could regulate satiety by increasing GLP-1 and PYY secretion from L cells in the distal gut. Supplementation of prebiotics containing fermentable polysaccharides increased plasma GLP-1 and PYY concentration in humans, while also increasing satiety and reducing hunger (102). In addition, myriad human and rodent studies have reported variable effects of fiber and indigestible carbohydrates on appetite and anorectic hormone release (103-105). A recent study found that β-glucan and fructooligosaccharide supplementation does not affect appetite or food intake, suggesting that a longer period of fiber consumption is needed to influence satiety (106). Some studies in healthy humans have shown that dietary fiber increase PYY_3-36_, yet no effect on satiety was reported (107), while others show that decreased food intake can occur independently of gut hormones in mice (108). Another study proposed that SCFAs, specifically acetate, crosses the blood–brain barrier and acts directly in the hypothalamic ARC to induce satiety (109). Considering these variable findings, it is possible that SCFAs act via multiple mechanisms to influence appetite, but whether the effect is largely dependent on gut hormones is yet to be determined.

CCK is released postprandially by I cells in the proximal gut, but unlike GLP-1 and PYY, it does not readily penetrate the blood–brain barrier, and therefore relies on the stimulation of CCK-A receptors on vagal afferents to elicit some of its satiety effects on the brain (110-113). There is some evidence showing the gut microbiota may alter the CCK system via microbial metabolite production. Mice with fructose malabsorption have increased density of I cells and *Cck* mRNA expression and in the ileum and cecum, which also was accompanied by increased levels of Actinobacteria, Bacteroidetes, and *Lactobacillus johnsonii* (*114*). Increased CCK levels appear to be attributed to changes in gut microbiota, as antibiotic-treated mice have much lower levels of *Cck* mRNA expression (114). Levels of cecal propionate is also increased in mice with fructose malabsorption (114). Propionate can trigger CCK gene expression in vitro, suggesting it may modulate CCK levels in mice, but mechanism driving this response remains unclear (114).

While there is strong evidence supporting the involvement of gut bacteria in appetite regulation, conflicting data suggest that the appetite suppression may only occur with specific dietary fiber used, as well as the duration of supplementation. Further research is required to clarify whether microbial metabolites are essential for regulating satiety in humans. Ultimately, the gut microbiome represents a critical factor in appetite control, with potential implications for obesity management.

## Host Metabolism

Whole-body metabolism involves the close coordination of several metabolically active tissues including the GI tract, skeletal muscle, brown adipose tissue, white adipose tissue, pancreas, adipose tissue, and liver, and release of postprandial (GLP-1, GIP, PYY, 5-HT, and CCK) and fasting (ghrelin, 5-HT) hormones (82)). The directionality of the metabolic disease–gut biome–EEC relationship is complex because metabolic diseases both effect and are affected by gut hormones, which are themselves effect and are affected by the gut biome. The complex integration of multiple distinct systems is reflected in the conflicting findings reported in the literature. For instance, the delivery of exogenous SCFAs like acetate, propionate, and butyrate can protect against diet-induced obesity by regulating gut hormone levels that control appetite in both human and mice (42, 115). Colonic delivery of propionate in humans stimulates GLP-1 and PYY release, leading to a reduction in weight gain and protection against adverse metabolic effects like insulin sensitivity (115). Similarly, butyrate, which has been shown to stimulate the release of GLP-1 and PYY from L cells (41, 46), enhances energy expenditure by promoting fat oxidation and activation of brown adipose tissue (116). However, while SCFAs appear to protect against diet-induced obesity, obese individuals and mice harboring an obese microbiome often have higher concentrations of SCFAs and a higher abundance of bacteria involved in SCFA biosynthesis (117, 118). Mice with obese microbiomes also appear to have an increased ability to harvest energy from their food (5). Because SCFAs are a well-known byproduct of additional calorie extraction, the higher SCFA levels observed may reflect this increase in calorie consumption. Indeed, some have suggested that high levels of SCFAs may be a consequence of obesity and not a cause, with obese individuals becoming resistant to the antiobesity effects of SCFAs over time (117, 119). It has also been suggested that alterations in SCFA levels is due to dysbiosis in the colon. This may help to explain the observed discrepancies, wherein SCFAs may retain their antiobesity effects when absorbed proximally to the colon, but these affects are negated by an overabundance of SCFA-producing bacteria in the colon.

Microbiome–EEC communication can also differentially influence the development of comorbidities associated with obesity, such as insulin resistance. For instance, while higher levels of SCFAs are associated with improvements in insulin sensitivity (120), obesity and insulin resistance are both correlated with an increase in bile acid synthesis and FXR expression (121-123). FXR inhibits proglucagon expression and GLP-1 secretion by reducing FFAR2 expression on L cells (124). Conversely, FXR deficiency protects against diet-induced obesity in mice, suggesting that FXR upregulation may contribute to the development of insulin resistance (72). Leveraging the bile acid signaling pathway using bile acid analogues also promotes GLP-1 secretion by inhibiting FXR, activating TGR5 and overall improving glucose regulation (125, 126). Altering the gut microbiome also alters the composition of the bile acid pool, leading to changes in these signaling pathways (127), and modulation of glucose and energy homeostasis (128, 129). Similarly, TLR signaling is important in the onset and progression of insulin resistance, specifically via lipopolysaccharide and other pathogen-associated molecular patterns (120, 130), but also in how it interacts with alterations to dietary lipid composition. For instance, an increase in saturated dietary lipids can directly influence microbiome composition, leading to a TLR-4–mediated increase in inflammation and insulin resistance (131). Conversely, the administration of exogenous metabolites (postbiotics) that act via TLRs can improve insulin sensitivity and glucose tolerance (132). While these studies did not directly link TLR activity to EECs, more recent research shows that polyunsaturated fatty acids, specifically docosahexaenoic acid, improve insulin resistance via an increase in SCFA production, PYY expression, and a reduction in epithelial damage (133). This suggests that EECs are likely to play an important role in TLR-mediated insulin resistance.

## Conclusions

The intricate relationship between the gut microbiome and EE cells is central to regulating various physiological processes, including metabolism, gut motility, and satiety. The microbiota's influence on EE cell function is primarily mediated through microbial metabolites, such as SCFAs, which activate specific receptors on EE cells to modulate hormone release and gut–brain communication. Additionally, bacterial signaling, via TLRs and other pattern recognition receptors, plays a pivotal role in maintaining proper EEC function and contributing to overall host health. This interaction between the microbiome and EEC has significant implications for metabolic diseases, such as obesity and diabetes, where dysbiosis and altered EEC signaling can exacerbate disease progression. The review highlights the complexity of these microbiome–EEC interactions, offering insight into how gut health is closely linked with systemic physiological processes.

Research in this area should focus on further unravelling the specific mechanisms by which different microbial metabolites and bacterial species regulate EEC function. Understanding the nuanced role of non-bacterial biomes, such as viruses and eukaryotes, in influencing EEC activity could also offer new perspectives on gut health. Moreover, studying how diet, lifestyle changes, and therapeutic interventions can modify the gut microbiome to improve EEC function could pave the way for novel treatments for metabolic and GI disorders. Investigating the potential for personalized microbiome-based therapies, where specific microbial strains are targeted or supplemented, holds promise in managing diseases linked to gut dysfunction, such as function dyspepsia and irritable bowel syndrome, as well as and systemic health. As research progresses, exploring these interactions could provide groundbreaking insights into the gut's influence on overall human health and disease management.

## Data Availability

Data sharing is not applicable to this article as no datasets were generated or analyzed during the current study.

## Abbreviations

5-HT: 5-hydroxytryptamine or serotonin
AGRP: agouti-related peptide
ARC: arcuate nucleus
ASBT: apical sodium-dependent bile acid transporter
CA: cholic acid
CCK: cholecystokinin
CDCA: chenodeoxycholic acid
DCA: deoxycholic acid
EC: enterochromaffin
EE: enteroendocrine
EEC: enteroendocrine cell
FFAR1: free fatty acid receptor 1 or GPR40
FFAR2: free fatty acid receptor 2 or GPR43
FFAR3: free fatty acid receptor 3 or GPR41
FFAR4: free fatty acid receptor 4 or GPR120
FXR: farnesoid X receptor
GI: gastrointestinal
GIP: glucose-dependent insulinotropic peptide
GLP: glucagon-like peptide
LCA: lithocholic acid
LPS: lipopolysaccharide
mRFP: monomeric red fluorescent protein
Olfr588: olfactory receptor 588
POMC: proopiomelanocortin
PYY: peptide YY
SCFA: short-chain fatty acid
TDCA: taurine conjugated deoxycholic acid
TGR5: Takeda G protein–coupled receptor 5
TLR: toll-like receptor
Trpa1: transient receptor potential ankyrin A1
Tph1: tryptophan hydroxylase 1
